# Sonographic Anatomy and Imaging of the Extracranial Component of the Hypoglossal Nerve (CNXII)

**DOI:** 10.1002/jmrs.70010

**Published:** 2025-07-18

**Authors:** Michelle Fenech, Jodie Gallagher, Laurelie R. Wishart, Clare Berry, Michael Foster‐Greenwood

**Affiliations:** ^1^ College of Clinical Sciences, School of Health, Medical and Applied Sciences Central Queensland University Brisbane Queensland Australia; ^2^ Royal Brisbane and Women's Hospital Brisbane Queensland Australia; ^3^ College of Clinical Sciences, School of Health, Medical and Applied Sciences Central Queensland University Melbourne Victoria Australia; ^4^ School of Medicine and Dentistry Griffith University Nathan Queensland Australia; ^5^ Office of the Chief Allied Health Practitioner, Metro North Allied Health Herston Queensland Australia; ^6^ School of Health & Rehabilitation Sciences The University of Queensland St Lucia Queensland Australia; ^7^ Herston Imaging Research Facility (HIRF) Brisbane Queensland Australia; ^8^ I‐Med Brisbane Queensland Australia

**Keywords:** ansa cervicalis, dysphagia, hypoglossal nerve palsy, radiation induced nerve fibrosis, tongue atrophy, ultrasound guided blockade

## Abstract

The hypoglossal nerve (HN) provides motor innervation to tongue muscles responsible for tongue movement, speech, mastication, swallowing, respiratory functions and management of oral secretions. Injury, compression, entrapment or lesions of the HN at any point along its path can result in HN palsy and subsequent dysphagia, dysarthria and tongue muscular atrophy. A combined imaging approach is required to investigate the HN and causes of HN palsy. Magnetic resonance imaging (MRI) and computed tomography (CT) imaging are used to investigate the intracranial HN and where it emerges in the upper neck. The extracranial HN can be assessed by sonographic imaging along with the muscles directly and indirectly innervated by the HN. Ultrasound imaging can be challenging without an appropriate understanding of the detailed relative anatomy of the HN and the muscles it innervates, the associated sonographic technique and sonographic appearances, all of which are outlined in this paper.

## Graphical abstract

The hypoglossal nerve (HN) provides motor innervation to tongue muscles responsible for tongue movement, speech, mastication, swallowing, respiratory functions and management of oral secretions. The intracranial component of the HN can be demonstrated on magnetic resonance imaging and the extracranial component can be demonstrated with sonographic imaging. Injury, compression, entrapment, or lesions of the HN can occur at any point along its path and cause HN palsy. Imaging, as outlined in this paper, can be used to identify HN changes.

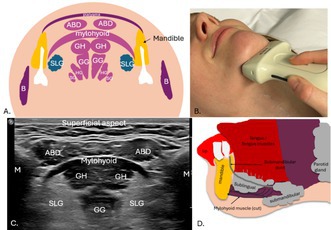

## 1 Introduction

The hypoglossal nerve (HN), the 12th cranial nerve (CNXII), travels from the brain to the upper neck and mouth region, providing motor innervation to tongue muscles [1]. It is responsible for tongue movement, speech, mastication, swallowing, respiratory functions and management of oral secretions [1, 2]. The infrahyoid strap muscles of the neck, which move the hyoid bone and thyroid cartilage during speech, swallowing and mastication, are indirectly innervated by the HN via the ansa cervicalis [1]. Pathology, injury, or entrapment of the HN can occur at any location along its intracranial and extracranial path, resulting in subsequent tongue paralysis, dysarthria (speech difficulties), dysphagia (swallowing difficulties), dry mouth and unilateral denervation and atrophy of tongue and infrahyoid muscles [2, 3].

HN lesions are defined by their location and can be classified as central (intracranial) and peripheral (extracranial); they can occur in isolation, at multiple points along the nerve path, or in association with other nerve palsies, all of which can confuse the clinical picture [1, 4]. The HN can be impacted by fibrotic tissue forming around the nerve from surgery or post‐radiation fibrosis, infection, lymphadenopathy, or other pathology compressing the nerve, tumour entrapping or infiltrating the nerve, tumours arising within the nerve itself, or injury from carotid and vertebral artery dissections [3]. Furthermore, anatomical variants in the neck, HN iatrogenic injury, neck trauma and neck pathology surrounding the HN can all be contributors to HN neuropathy and palsy [3, 5, 6, 7]. Surgeries such as carotid endarterectomy, neck dissection, or tonsillectomy are common surgical causes of HN iatrogenic injury [3]. Imaging plays a central role in the diagnosis of a structural cause of HN palsy and guiding management of HN injury, entrapment, or pathology.

A multi‐modality approach to imaging the entire HN is required as the intra‐cranial and extra‐cranial HN can be demonstrated effectively by different imaging modalities [8]. Computed tomography (CT), magnetic resonance imaging (MRI) and/or 18F‐fluordeoxyglucose positron emission tomography CT (PETCT) can be used to image the intracranial HN and its surrounding bony and soft tissues, and the muscles it subsequently innervates [2]. Ultrasound imaging provides excellent spatial resolution to directly image the extracranial HN, and can also provide quick, non‐invasive, static and dynamic imaging of its innervated muscles [9].

Underappreciation of the sonographic relative anatomy of the HN, the structures it innervates, and the sonographic technique can result in it being overlooked when in the ultrasound imaging field of view during routine neck ultrasound examinations. There is currently underutilisation of direct sonographic imaging of the HN. As ultrasound imaging has a much smaller field of view relative to CT, MRI and PETCT, and relative landmarks which signpost the position of the HN, used in larger field of view imaging are not readily identifiable. Hence a detailed understanding of anatomy is required to sonographically image the HN. Additionally, the small HN can appear sonographically inconspicuous, as it can blend with surrounding tissues with altered angles of insonation, so a proficient sonographic technique to image the HN is required.

An outline of anatomic landmarks that can signpost the location of the extracranial HN, the sonographic technique to allow it to be effectively imaged with ultrasound is required as there is currently a paucity of literature on this topic. This paper addresses this gap. As a multi‐modal approach to imaging the HN is required, the path and imaging of the intracranial HN is required to be appreciated by those undertaking sonographic examinations and briefly discussed.

## 2 The Intracranial Hypoglossal Nerve

The HN is part of the posterior group of cranial nerves which also includes the glossopharyngeal nerve (CNIX), vagus nerve (CNX), spinal accessory nerve (CNXI) [10]. Situated in the posterior cranial fossa, they all emerge from the medulla oblongata [11]. The intracranial components of these nerves are all best demonstrated with MRI [8]. The intracranial component of the HN has medullary, cisternal and intracanalar (skull base) segments [8].

**FIGURE 1:**
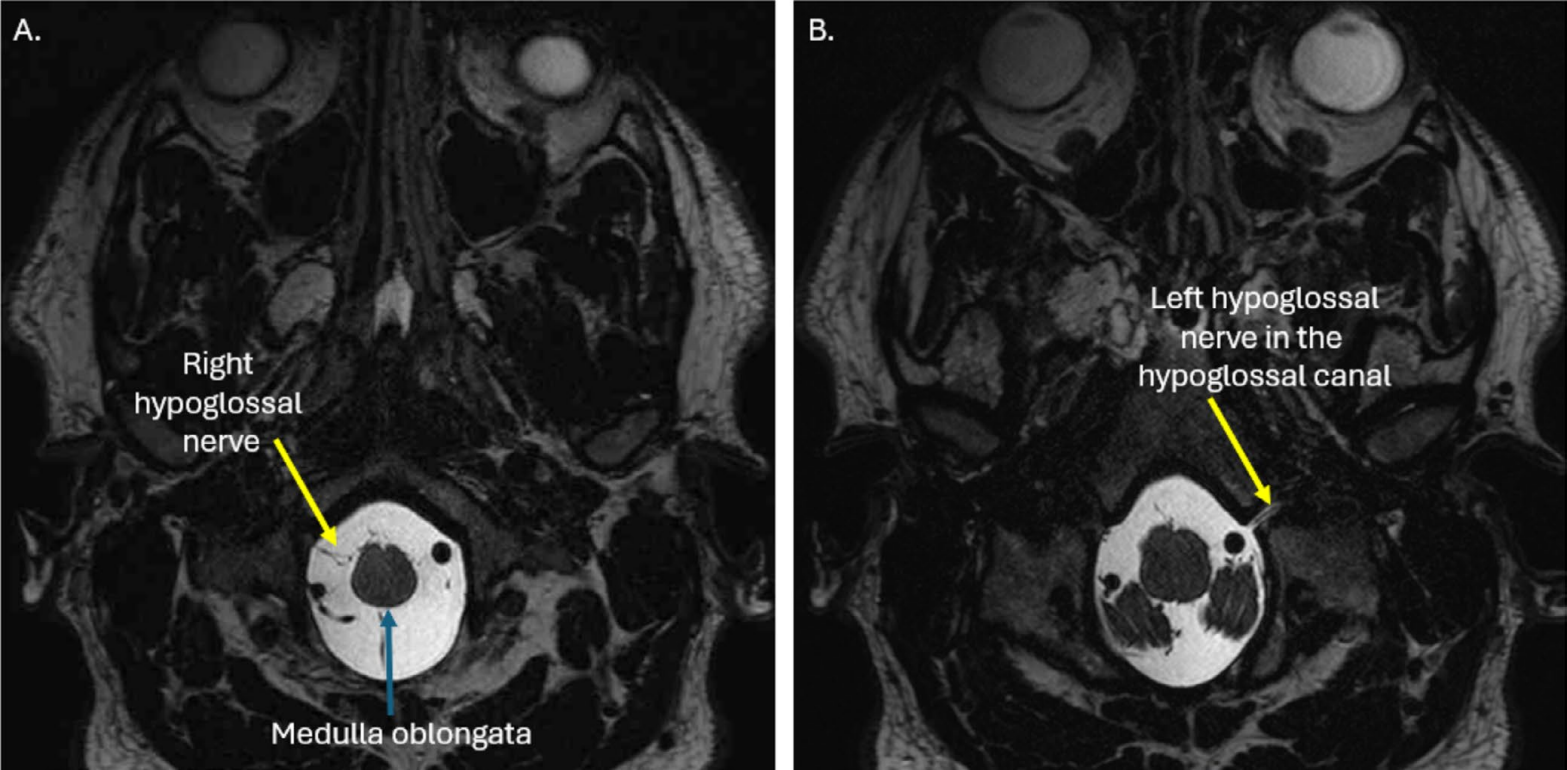
Proximal path of the hypoglossal nerve (HN). (A, B) The intracranial component of the hypoglossal nerve demonstrated on T2 fat sat, SPACE, axial magnetic resonance (MR) images (slices: 0.5 mm thick). (A) The HN arises from the medulla oblongata, posterior to the clivus and close to the vertebral arteries. (B) The HN subsequently courses through the hypoglossal canal. The HN is small (very thin).

The HN arises as a multitude of rootlets from the medulla oblongata in the pre‐olivary groove [3]. The HN rootlets pass anteriorly through the pre‐medullary cistern, near the vertebral artery, and the posterior inferior cerebellar artery (PICA) [8, 12]. The rootlets traverse the bony hypoglossal canal between the occipital condyle and the jugular tubercle of the occipital bone, where they converge to form a single HN, appreciable on axial MRI [3, 8]. The HN travels antero‐obliquely to emerge from the skull into the upper neck [3, 8, 12, 13] (Figure 1).

Due to their small size, imaging of the posterior cranial nerves using conventional MRI is not reliable [11]. Studies reporting optimal MR imaging of the HN and the size of the intracranial HN are scarce [4]. The diameter of the intracranial HN measured via a cadaveric study is approximated at 1 mm [14]. Volumetric MR imaging is required to demonstrate the small size and oblique path of the intracranial HN [4]. Intracranial paired right and left HNs should appear symmetrical in size where isotropic voxels of a maximum size of 1.2 × 1.2 × 1.2 mm^2^ are used [4, 11, 15].

CT imaging does not directly demonstrate the HN; however, it is useful for demonstrating any nearby bone destruction of the hypoglossal canal or associated traumatic bone fractures which can transect, irritate, or entrap the HN [4]. Compromise of the intracranial HN can be further affected by intracranial haemorrhage, ischaemia, abscess formation, malignancies and other space‐occupying lesions all demonstrable on CT [4]. PETCT is useful for identifying pathological lesions within or adjacent to the intracranial HN, within or around the hypoglossal canal and foramen and highlighting neck extension of pathology [16]. Additionally, vascular structures and anomalies around and within the hypoglossal canal, which may impact the HN, can be identified with CT and PETCT [4].

## 3 Sonographic Imaging and Anatomy of the Extracranial Hypoglossal Nerve

The extracranial HN can be difficult to demonstrate with MRI due to motion artefacts from swallowing, tongue movement and flow in vessels, which degrades image quality [4]. In the neck, CT and MRI can be used to demonstrate the tissues around the HN and denervation of tongue and neck structures as a result of HN palsy; however, they will not directly demonstrate the fascicular nature of the extracranial HN [2, 3, 17, 18]. Hence, ultrasound imaging, due to its high spatial resolution, can be used to directly image the extracranial HN, its surrounds, and identify any evidence of pathology, injury, or entrapment, and distal denervation changes [3]. Dynamic imaging can also be used to demonstrate any temporal entrapment of the HN [19]. The extracranial HN is an important surgical landmark; hence, sonographic imaging of this nerve can be used not only to diagnose HN injury or pathology but also to aid surgical planning [20].

MR and CT imaging of the intracranial HN, if previously conducted, should be reviewed prior to an ultrasound examination to ensure a multisegmented approach to HN imaging [2]. Dynamic real‐time ultrasound imaging allows movement of the HN to be visualised to ensure it is not tethered, as well as motion of the muscles surrounding the HN and those innervated by the HN [9]. Ultrasound imaging can be additionally used to localise the HN during ultrasound guided procedures of the neck, such as biopsies of nearby lymph nodes, and to avoid iatrogenic injury, ensuring the HN is not within the needle trajectory [21].

Sonographic imaging of the HN requires both long and short axis imaging [2]. In long axis, the HN should appear to have relatively parallel borders with a fascicular echotexture where hypoechoic fascicles are surrounded by echogenic interfascicular perineurium, internal epineurium and an outer layer of echogenic epineurium [3]. On short axis sonographic imaging, the unaffected HN should appear round and demonstrate the classic honeycomb neural appearance [22]. The HN is assessed for its continuity and focal, fusiform or diffuse thickness and echogenicity changes [22]. Nerve thickening can indicate intrinsic nerve lesions, swelling around entrapment, irritation and scarring due to injury, and areas of thinning can indicate compression due to entrapment and areas of partial transection [9]. Discontinuity of the nerve indicates complete transection [23].

The sonographic cross‐sectional area (CSA) of the extracranial HN in healthy volunteers is reported as 1.9 mm^2^ at the postero‐lateral rim of the mylohyoid muscle and 2.1 mm^2^ where the HN crossed the external carotid artery (ECA) [3]. However, true CSA measurements of this nerve can be difficult to replicate due to the oblique path of the HN and difficulty maintaining appropriate transducer angulation, which can affect true short‐axis imaging [3]. When not imaged in true short‐axis, the HN CSA measurements can be overestimated [9].

Diameter measurements of the HN thickness (measured in mm) from long axis imaging can be an easier and potentially more reliable way to obtain nerve measures, and documentation of quantitative focal thickness changes; however, studies reporting sonographic thickness measures of the unaffected HN and the reliability of these measures are lacking. Typically, nerve sizes are sonographically compared to the contralateral unaffected side, and studies reporting contralateral symmetry of HN size are also required.

For sonographic imaging of the extracranial HN to be conducted, the patient is positioned supine, neck extended, and face turned to the opposite side of imaging, allowing maximal transducer access to areas around the mandible [3]. To undertake high‐resolution sonographic imaging of the HN, high‐frequency linear transducers ≥ 12 MHz are required [24]. A larger footprint transducer allows relative anatomy to be appreciated, although smaller footprint hockey stick transducers allow easy manoeuvring around the angle of mandible [21].

Factors that can limit the sonographic examination of the HN can include patients with large necks, as increased neck adipose tissue can degrade image quality [21]. Short necks can impede transducer movements, making ultrasound imaging difficult and beard hair can trap air and impede sound transmission [9]. Patients who have had neck radiotherapy may have fibrotic change, which can attenuate and scatter the ultrasound and degrade sonographic image quality [25, 26]. Additionally, posterior acoustic shadowing can occur deep to scars from previous neck surgery, limiting HN visualisation [25].

When conducting a sonographic examination of the extracranial HN, the approach can be subdivided into assessing the nerve at different segments and orientations: (1) descending and (2) horizontal [8].

## 4 Descending Portion of Extracranial Hypoglossal Nerve

**FIGURE 2:**
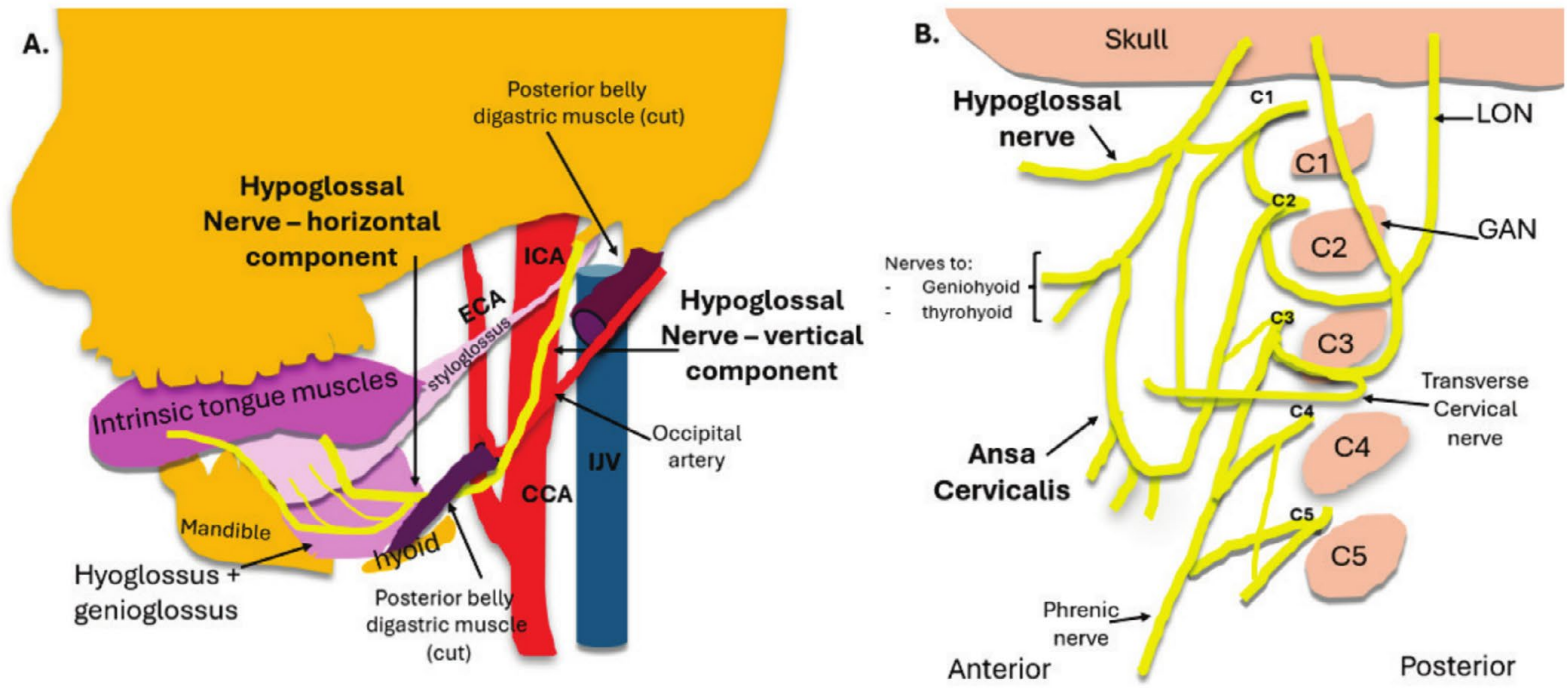
Course of the extracranial portion of the hypoglossal nerve (HN). (A) Path of the HN relative to other structures. (B) Interaction of the HN with other cervical nerves. CCA, common carotid artery; ECA, external carotid artery; GAN, great auricular nerve; ICA, internal carotid artery; IJV, internal jugular vein; LON, lesser occipital nerve.

When obtaining sonographic imaging, the path and surrounds of the HN must be appreciated to allow appropriate transducer placement. Vascular landmarks aid in signposting the position of the descending HN and the ICA is a useful consistent landmark [3]. In the upper neck, the HN descends, and posterior to the styloid process of the temporal bone, anterior to the transverse process of C1, along with the internal carotid artery (ICA) and vagus nerve (CNX) [2, 8]. This most proximal portion of the extracranial HN is most challenging to image sonographically, and if a lesion is suspected at this site, multi‐modality imaging is required [3]. The HN lies initially deep to the upper sternocleidomastoid (SCM) muscle, an easy to identify sonographic landmark. In the upper neck, the HN communicates with a branch of the anterior ramus of C1, which should not be confused sonographically for the HN [8, 12] (Figure 2).

**FIGURE 3:**
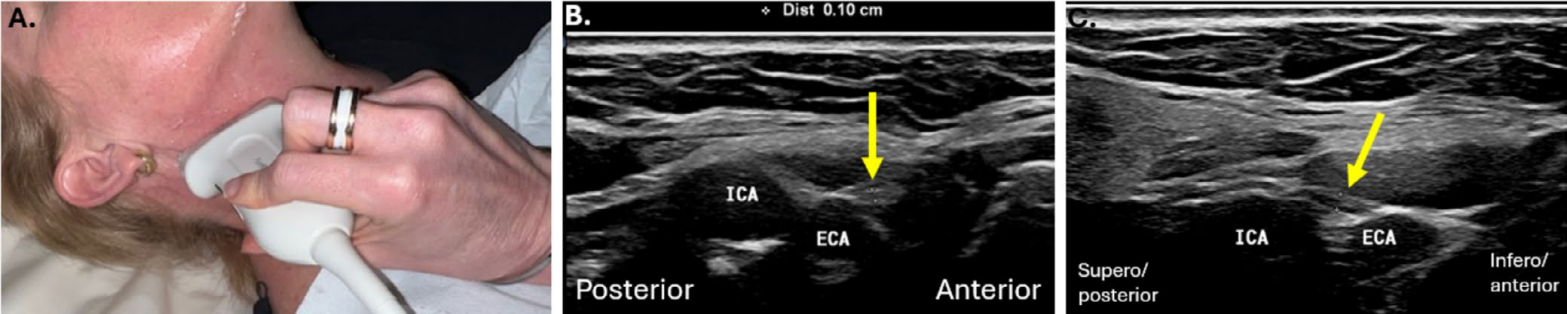
(A) Transducer positioning for imaging where the hypoglossal nerve changes from its horizontal to vertical component. Sonographic imaging of the posterior horizontal portion of the hypoglossal nerve (B) and the start of the vertical component of the hypoglossal nerve (C).

The HN position alters relative to the ICA throughout its course from proximal to distal. Superiorly in the neck, the HN sits deep to the ICA, and as it courses more disto‐anteriorly, it courses to sit between the ICA and internal jugular vein (IJV) [2]. Posterior to the ramus of the mandible, the HN continues to pass in a rotatory course to sit anterior to the ICA, coursing antero‐inferiorly, superficial to the anteriorly placed occipital artery, and subsequently the occipital artery origin from the external carotid artery (ECA), which is located just superior to the hyoid bone [1, 12]. Sonographically, the descending HN is easier to identify more distally, where it can then be tracked proximally in short axis to the region of the mastoid process and styloid process of the temporal bone [1, 3] (Figure 3).

## 5 Horizontal Portion of the Extracranial Hypoglossal Nerve

Around the angle of the mandible, and the inferior margin of the posterior belly of the digastric muscle and anterior margin of the SCM, the HN becomes more horizontal in orientation, coursing anteriorly towards the tongue [1, 8]. During its horizontal course, surrounding muscles are useful in signposting the position of the HN with sonographic imaging [2]. Where it becomes horizontal in orientation, the HN gives rise to the anterior (superior/descending) root of the ansa cervicalis (AC) which innervates three of the infrahyoid muscles: the sternohyoid, sternothyroid and omoyhyoid [1]. The AC roots will be observed to course inferiorly, where the HN courses anteriorly [2].

The HN passes anteriorly between the hyoid bone and the middle tendon of the digastric muscle, a doing so superficial to the hyoglossus muscle, an important sonographic and surgical landmark [8]. The hyoglossus muscle separates the deeper lingual artery from the superficial HN [20]. The lingual artery should be confirmed with colour Doppler and not confused for the HN or the more superiorly positioned lingual nerve [27]. The lingual artery, typically the second branch of the ECA, is the primary arterial supply to the tongue and floor of mouth [20]. Throughout its distal course, the HN remains superficial to the lingual artery [1]. The lingual nerve, a branch of the mandibular branch of the trigeminal nerve, provides sensory innervation to the anterior two‐thirds of the tongue and sits approximately 2 cm superior to the HN [20].

**FIGURE 4:**
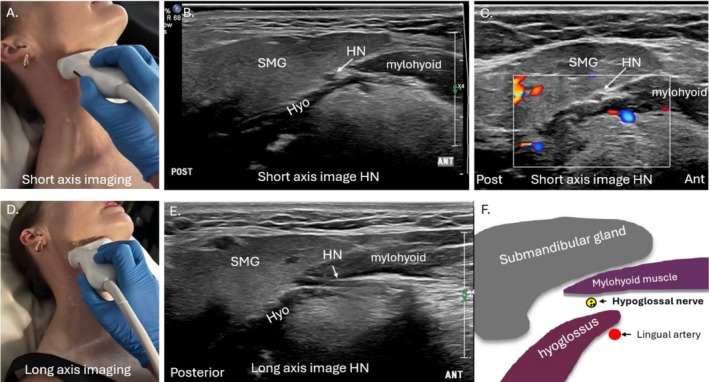
Short and long axis imaging of the hypoglossal nerve (HN). (A) Transducer positioning for short axis imaging of the HN. (B) Sonographic short axis image of the HN and lingual artery (LA). (C) Colour Doppler image to demonstrate colour fill in the LA and confirm its position. (D) Transducer positioning for long axis imaging of the HN. (E) Sonographic long axis image of the HN. (F) Diagram to demonstrate the relationship of the submandibular gland (SMG), mylohyoid and hyoglossus muscles and HN and LA in short axis. ANT, anterior aspect of image; Hyo, hyoglossus muscle; POST, posterior aspect of image.

As the HN courses further anteriorly, it will course deep to the mylohyoid muscle. The HN is easiest to initially localise sonographically at this point, in the submental space, between the mylohyoid and hyoglossus muscles [28]. The nearby submandibular (Warton's) duct should also not be confused for the HN; it runs parallel and superior to the HN [8, 20]. The transducer is placed under the chin in a coronal‐oblique plane [21]. This ‘landing point’ of where the transducer is placed on the skin, allows short axis imaging of the HN, which is made conspicuous where it sits in a fatty plane between the mylohyoid and hyoglossus muscles [21]. This aligns with a position deep to the anterior border of the submandibular salivary gland, easily identified sonographically, and easily correlated with MRI if required (Figure 4).

Once sonographically identified between the mylohyoid and hyoglossus muscles, the HN can be tracked antero‐medially deep to the mylohyoid muscle before coursing cranially to innervate the tongue muscles [3, 29]. Transducer angulation adjustments are required when tracking the HN, as it can become isoechoic with surrounding tissues, rendering it sonographically inconspicuous due to anisotropic phenomena (Video S1) [3].

Long axis sonographic imaging of the HN is also performed and in the anterior submandibular region, the transducer can be rotated as close to 90° as possible to a sagittal oblique plane, parallel to the body of the mandible [9]. The HN can be observed to traverse through the plane between the superficial mylohyoid muscle and deeper hyoglossus muscle in long axis, where focal thickening or thinning of the nerve or echogenicity changes are easily assessed, and nerve thickness measurements can be performed if required [21].

## 6 Anatomy of Structures Related to the Hypoglossal Nerve Important for Imaging

Denervation of the intrinsic and extrinsic tongue muscles directly innervated by the HN and the infrahyoid muscles, indirectly innervated by the HN via the ansa cervicalis, can occur with HN palsy and should be assessed with imaging [2]. The suprahyoid muscles additionally signpost the position of the HN during a sonographic assessment, and hence, their sonographic anatomy should be understood.

### 6.1 Tongue Muscles

There are two groups of tongue muscles, the extrinsic and intrinsic muscles, both innervated by the HN [13]. The intrinsic tongue muscles, all innervated by the HN, include the superior and inferior longitudinales, transversus and verticalis [20]. They change the three‐dimensional shape of the tongue and allow tongue shortening, narrowing and curving but are not routinely individually identified on imaging [30]. The dorsal, superior aspect of the tongue, superficial to the superstructure, is lined with a thick mucosa and papillae anteriorly and lymphatic tissue posteriorly, usually assessed by visual inspection [30]. It should be noted that the ventral tongue refers to its inferior aspect [20] (Table 1).

**TABLE 1:** Extrinsic and intrinsic tongue muscles and their origins, insertions and actions.

Muscle	Origin	Insertion	Action
Extrinsic muscles			
Genioglossus	Superior mental spine mandible	Dorsum tongue, lingual aponeurosis, hyoid bone body	Depresses, protrudes and deviates tongue
Hyoglossus	Body and greater horn of hyoid bone	Infero‐anterior parts of lateral tongue	Depresses and retracts tongue
Styloglossus	Anterolateral styloid process and stylo‐mandibular ligament	Inferior longitudinal and hyoglossus muscles	Retracts and elevates lateral aspects of tongue
Palatoglossus^a^	Palatine aponeurosis of soft palate	Lateral tongue margins	Elevates root of tongue and constricts tongue isthmus
Intrinsic muscles			
Superior longitudinal (superior longitudinales)	Posterior tongue submucosa and lingual septum	Anterolateral tongue	Retracts and broadens tongue, elevates tongue base
Inferior longitudinal (inferior longitudinales)	Tongue root and body of hyoid bone	Apex of tongue	Retracts and broadens tongue and lowers tongue apex
Transverse muscle (transversalis)	Lingual septum	Lateral tongue	Narrows and elongates tongue
Vertical muscle (verticalis)	Tongue root and genioglossus muscle	Lingual aponeurosis	Broadens and elongates tongue

^a^ Palatoglossus muscle is innervated by the vagus nerve. All other tongue muscles are innervated by the hypoglossal nerve.

**FIGURE 5:**
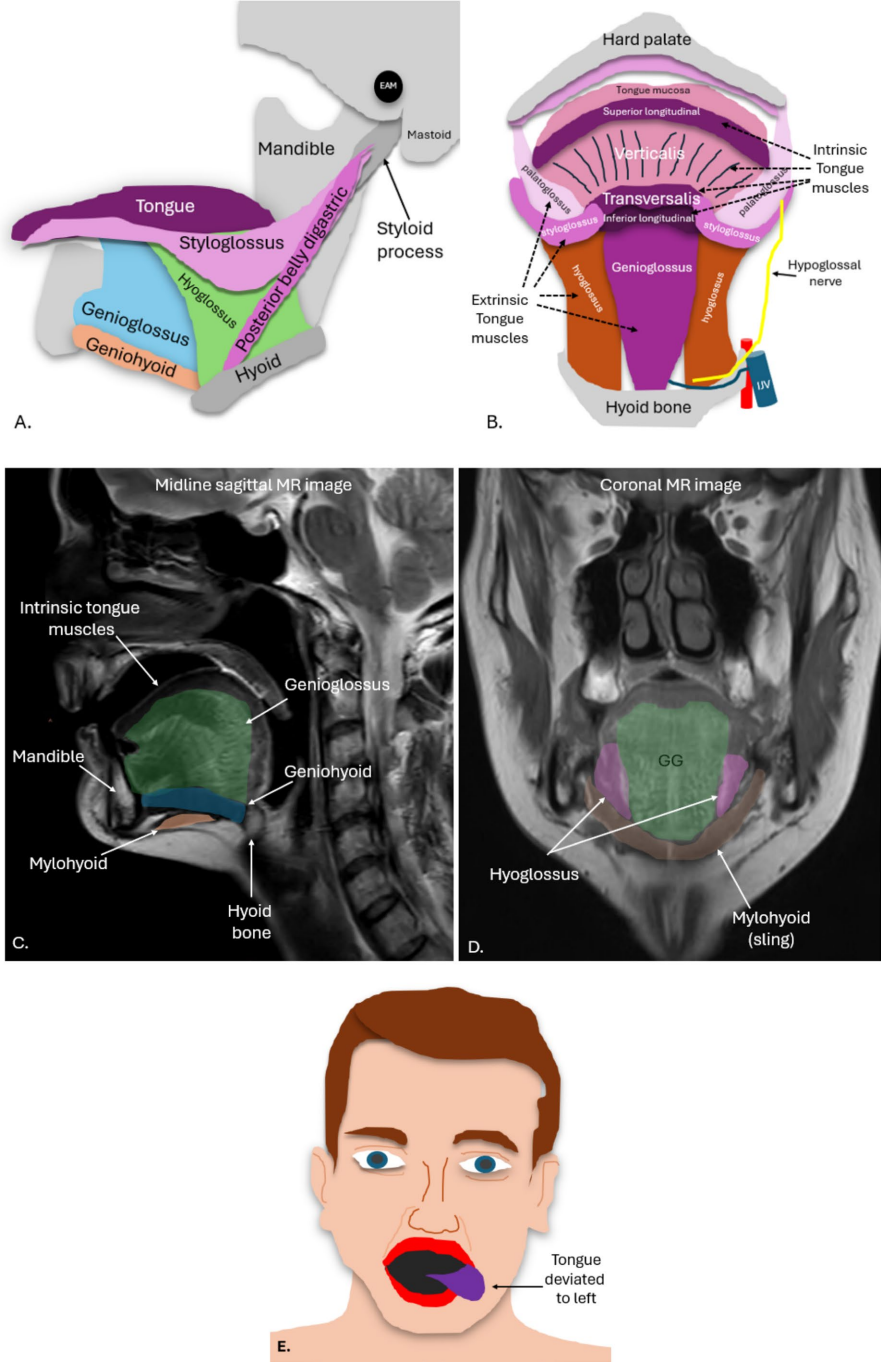
Tongue muscles. (A) Parasagittal diagram of extrinsic tongue muscles. (B) Coronal diagram of intrinsic tongue muscles. (C) Mid‐sagittal magnetic resonance (MR) image of the mouth region outlining key bones and muscles. (D) Coronal MR image demonstrating the relationship of the genioglossus (GG), hyoglossus and mylohyoid muscles. (E) Left hypoglossal nerve palsy patient may present with deviation of the tongue to the left. EAM; external auditory meatus, IJV; internal jugular vein.

The extrinsic tongue muscles include the genioglossus, hyoglossus, styloglossus and palatoglossus, which move and manipulate the tongue [2, 30]. The small palatoglossus muscle (innervated by CNX, not the HN) connects the soft palate to the tongue and pulls the tongue towards the soft palate, which aids in eating [20] The styloglossus and the palatoglossus muscles are more challenging than others to visualise sonographically but lie immediately lateral to the geniohyoid muscle [21]. The styloglossus muscle draws the tongue upward [30]. The genioglossus is the largest muscle and is easily identified sonographically when the transducer is placed under the chin; it protrudes the tongue and deviates it to the opposite side, and its movement is easily assessed with dynamic ultrasound imaging [20]. The thin hyoglossus muscle attaches the hyoid bone to the tongue, retracts the tongue, and depresses its sides and is an important sonographic landmark for identifying and assessing the HN, and its motion can also be assessed dynamically [30] (Figure 5).

Anatomical variations of the HN and the muscles it innervates can occur [1]. Further muscles including the sternocleidomastoid, stylohyoid, digastric, or mylohyoid muscles can all variably receive innervation from the HN [7]. Hence, unusual presentations of denervation of any of these muscles may be an indication for HN imaging [1].

### 6.2 Suprahyoid Muscles

The suprahyoid muscles include the digastric, mylohyoid, geniohyoid and stylohyoid muscles and they form the floor of the mouth, inferior to the tongue [20]. These muscles are used as sonographic landmarks to signpost the position of the HN and aid in describing the location of any HN or floor of the mouth pathology that can encase, invade, or entrap the HN [17, 31].

**FIGURE 6:**
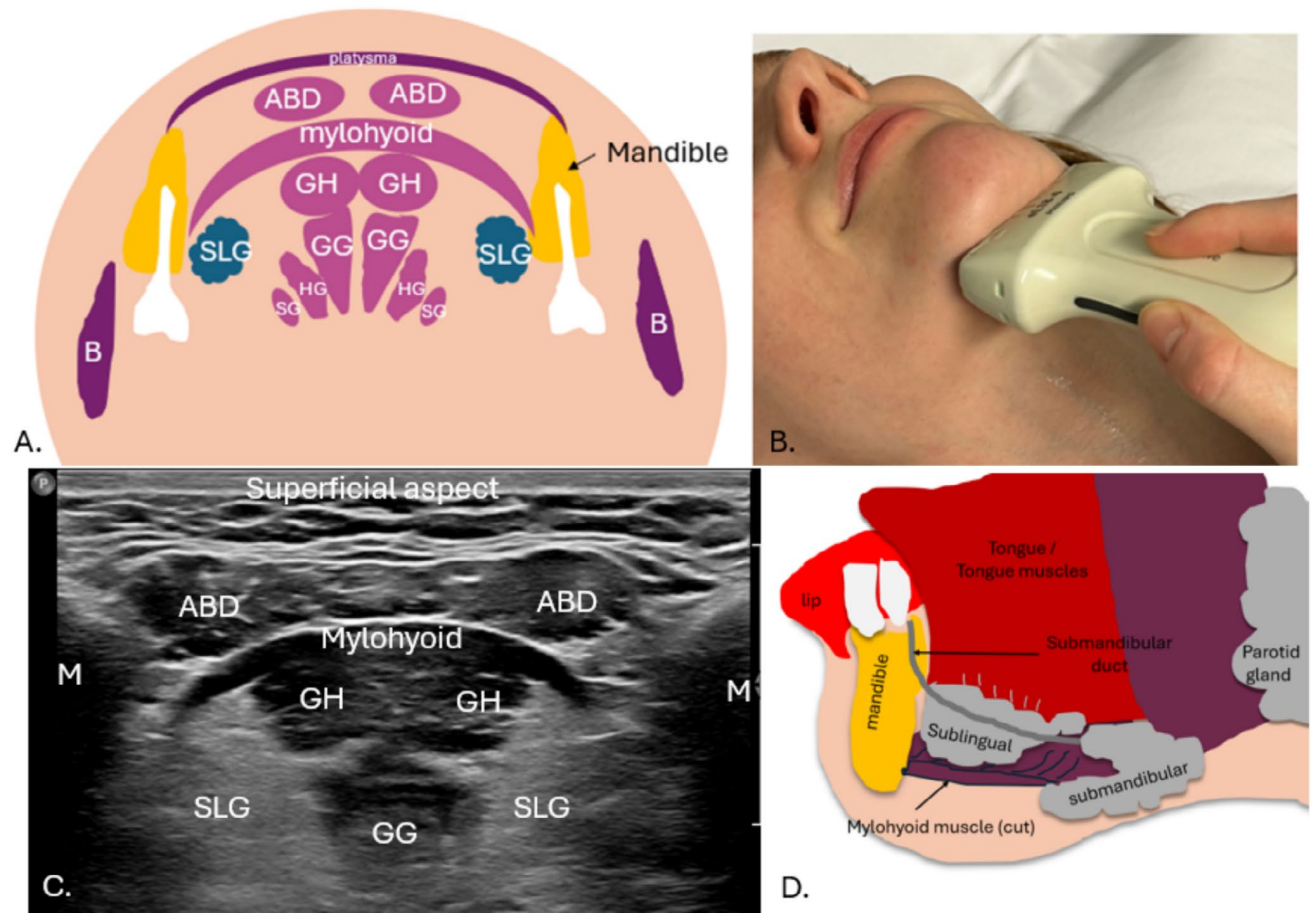
Floor of mouth musculature. (A) Coronal diagram of floor of mouth musculature. (B) Transducer positioning to coronally image the floor of mouth musculature. (C) Coronal ultrasound image of the floor of the mouth. (D) Para‐sagittal diagram of the mylohyoid muscle relative to the salivary glands. ABD, anterior belly of digastric muscle; B, buccinator muscle; GG, genioglossus muscle; GH, geniohyoid muscle; HG, hyoglossus muscle; M, mandible; SG, styloglossus muscle; SLG, submandibular gland.

The paired mylohyoid muscles form a sling‐like structure in the submental and submandibular region acting as a supportive diaphragm of the floor of the mouth and form a key sonographic landmark for identifying the HN, which is located deep to this muscle [20, 31]. The mylohyoid muscles sit deep to the anterior bellies of the digastric muscle [17, 21] and separate the sublingual space from the submandibular space; important for containing the spread of infections such as dental abcesses [20, 31]. Each mylohyoid muscle attaches the medial aspect of the mandible body to a midline raphe which extends from the mandibular symphysis to the hyoid bone [29]. The mylohyoid muscles additionally extend posteriorly from the mandibular symphysis at the level of the last molar tooth [17, 20, 31]. The HN distal branches course alongside and inferior (superficial) to the sublingual glands, which sit deep to the mylohyoid muscle [1, 17, 31] (Figure 6).

The free posterior border of the mylohyoid muscle is a landmark to define the deep and superficial submandibular gland components on imaging [17]. Mylohyoid muscle defects can occur, unilaterally or bilaterally, referred to as mylohyoid boutonnieres [20, 31]. Defects can result in herniated fat and salivary tissue, mostly from the sublingual glands, but also submandibular glands, plunging ranulas (benign mucus retention cysts), abscesses and malignancies which can subsequently entrap the HN leading to HN palsy [31, 32, 33].

The HN follows the path of the posterior belly of the digastric muscle as it travels in the upper neck, deep and inferior to it at different points along its path; hence, this muscle is a key landmark for determining the HN position. It is also a landmark used for submandibular gland surgeries and level I neck dissections [20]. The anterior and posterior bellies of the digastric muscles are separated by an intermediate tendon [20, 21]. The posterior belly attaches the mastoid notch of the temporal bone to the intermediate tendon, which attaches to the hyoid bone; the anterior belly attaches to the mandible [17].

The geniohyoid muscles sit between the mylohyoid and genioglossus muscles and are also a good sonographic landmark for identifying the genioglossus muscles for dynamic assessment of motion and tracking the HN and its genioglossal distal branches [21]. Originating from the mental spine, inferior to the origin of the genioglossus muscles, the geniohyoid muscles insert onto the body of the hyoid bone, reinforcing the floor of the mouth [20]. The stylohyoid muscles are often underappreciated on sonographic imaging; they sit in the parasagittal plane, extending from the styloid process of the temporal bone to the hyoid bone, superior and posterior to the hyoglossus muscles, anteromedial to the posterior belly of the digastric and lateral and posterior to the styloglossus muscle [17]. The stylohyoid muscle can be used as an imaging landmark, as the proximal extracranial HN passes deep to this muscle and the posterior belly of the digastric [2]. Where the HN passes the stylohyoid muscle, the HN can potentially become entrapped [29].

### 6.3 Infrahyoid (Strap) Muscles and Ansa Cervicalis

The HN provides innervation indirectly to the infrahyoid strap muscles via a nerve loop called the ansa cervicalis (AC) [1]. The infrahyoid or strap muscles consist of four paired muscles which connect the hyoid bone to the sternum, larynx and scapula; the deeper sternothyroid and thyrohyoid muscles and superficial omohyoid and sternohyoid muscles [21]. They can be easily assessed with static and dynamic sonographic imaging when HN entrapment is suspected [32]. Chronic denervation muscle changes such as muscle atrophy and fatty infiltration will be demonstrated sonographically by smaller muscle volume and increased echogenicity [2] (Video S2).

**FIGURE 7:**
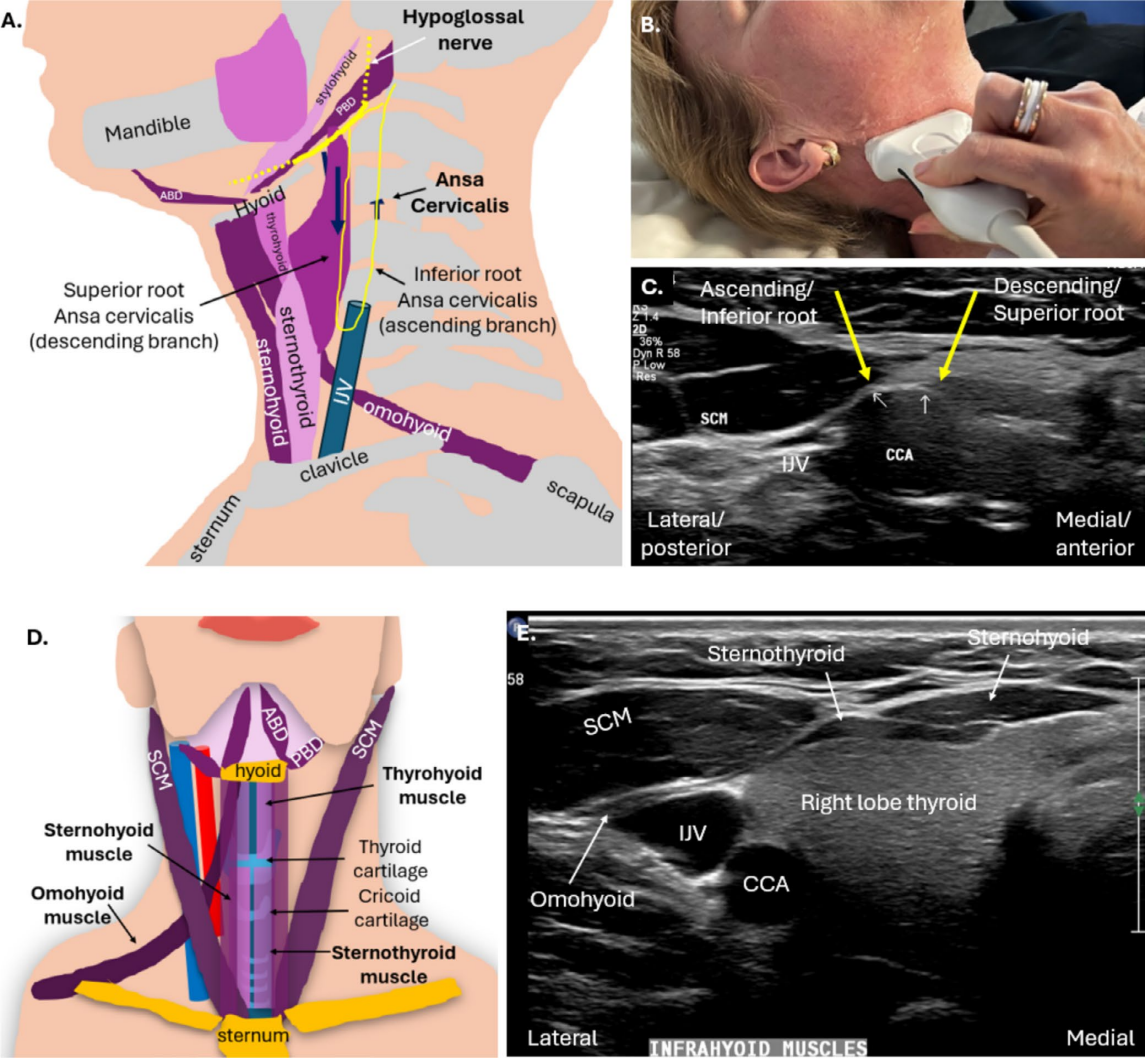
Anatomy of the ansa cervicalis and infrahyoid (strap) muscles. (A) Diagram of relative anatomy from lateral approach; the sternocleidomastoid muscle has been removed (and overlies structures of the lateral neck). (B) Transducer positioning for short axis imaging of the ansa cervicalis. (C) Short axis image of the ansa cervicalis roots (yellow arrows) overlying the common carotid artery (CCA). (D) Diagram of the anterior neck demonstrating the four strap muscles: Sternohyoid, omohyoid, thyrohyoid and sternothyroid. (E) Transverse sonographic image of the right side of the neck at the right lobe of the thyroid outlining some of the strap muscles. ABD, anterior belly digastric muscle; CCA, common carotid artery; IJV, internal jugular vein; PDB, posterior belly digastric muscle; SCM, sternocleidomastoid muscle.

The AC is composed of two roots: superior (anterior) and inferior (posterior) [2, 8]. The anterior AC (descending) root joins with the HN [13]. It branches and courses inferiorly, superficial to the carotid sheath [28]. The course of the posterior AC root is more variable [28]. The AC is sonographically imaged in conjunction with the HN, and should be differentiated from it; the AC sits inferior to the HN [28] (Figure 7).

Collectively, the suprahyoid and infrahyoid muscles control the position of the hyoid bone and are important for speech, swallowing and the movement of the larynx [34]. Infrahyoid paralysis, due to damage to the HN and AC, can cause swallowing difficulties, a hoarse voice and throat tightness [34].

## 7 Clinical Assessment of the Hypoglossal Nerve

The HN can be affected by a variety of conditions, pathologies, entrapment and injury and can result in HN palsy or paralysis [1]. An appreciation of how the HN is clinically assessed is required when imaging the HN and investigating HN palsy. Symptoms of HN palsy include tongue fasciculations and tongue paralysis of varied degrees, which result in a scalloped appearance of the tongue, and when chronically affected, cause unilateral tongue atrophy [2]. Tongue fasciculations can be caused by and are a characteristic symptom of HN damage in motor neuron disease [7]. Tremors of the tongue can also occur in cases of alcoholism and parkinsonism [1]. HN palsy, resulting in poor tongue function and decreased tongue strength, can lead to accumulation of saliva in the oral cavity, as well as severe functional deficits related to speech and swallowing, which limit the quality of life [5, 7]. Patients with HN pathology can present clinically with dysphagia, dysarthria and tongue muscular atrophy [7]. They can demonstrate tongue dysfunctions such as tongue deviation, weakness, or fasciculation [7].

Clinical oromotor assessment of both the right and left HNs involves examining the tongue strength, bulk and dexterity/range of motion, to make sure it is not weak, atrophied, moving abnormally or impaired [13]. The tongue is assessed in the resting and moving states where the patient is requested perform movements twice, once slowly and once rapidly [5]. Movements include poking their tongue out, moving their tongue in and out of their mouth, moving it from side to side and up and down [5]. The patient is also asked to elicit speech sounds such as ‘t’, ‘d’, ‘l’ and ‘s’ sounds, including rapid alternating movements (didaochokinesis) [35]. The strength of the tongue is tested by asking the patient to force their tongue to the side of their cheek, whilst the examiner tries to move the tongue with their finger or a tongue depressor [36].

Clinical examination is important for establishing the impact of HN palsy on tongue function, diagnosing functional deficits, potential rehabilitation planning, and is particularly useful when paired with more thorough instrumental assessment such as fibreoptic endoscopic evaluation of swallowing and fluoroscopic swallow studies [5]. However, these assessments cannot localise with accuracy the site and extent of nerve pathology or damage, which is where imaging plays an important role [2].

## 8 Pathology and Injury to the Hypoglossal Nerve: Causes and Structural Change

Imaging evaluation of patients with HN palsy can be used to identify HN structural change which can occur both intracranially (at the intra‐axial, cisternal and skull base) and extracranially [2]. At different points along its path, anatomical variants, trauma including HN iatrogenic injury, surrounding neck pathology and direct HN pathology can all be causes of HN neuropathy [1]. Compression/entrapment of the HN results in a focal thickness decrease and associated swelling on one or both sides of the compressed region; this can be encountered both intra and extra‐cranially [2]. HN neuroma formation due to injury results in a focal neural increase in thickness or cross‐sectional area [2].

Anatomical variants causing HN entrapment include carotid‐vertebrobasilar anastomosis, elongation of the styloid process, or ossification of its associated ligaments and tendons [1]. Carotid‐vertebrobasilar anastomoses can occur in conjunction with a persistent hypoglossal artery between the cervical segment of the ICA (level C1–3) and often require demonstration via MRI or CT [1]. Elongation of the styloid process of the temporal bone, or ossification of the stylohyoid ligament can cause concurrent compression of the ICA and HN resulting in Eagle syndrome, characterised by anterolateral neck pain radiating to the ear, syncope and transient ischaemic attacks [1, 37, 38].

Iatrogenic HN injury causes include surgery, IJV cannulations and post radiation fibrosis [7]. Surgery including carotid endarterectomy, neck dissection, tonsillectomy, neck biopsies, tooth extractions and cervical spine surgery can result in iatrogenic HN injury [1, 3]. HN palsy can also occur secondary to direct laryngoscopy, trans‐oral intubation, use of a laryngeal mask, tooth extractions and cervical spine surgery [1].

Injury or compression to the ipsilateral HN and recurrent laryngeal branch of the vagus nerve (RLBVN) results in concomitant palsy to these nerves, where patients can present with the clinical condition Tapia's syndrome [39, 40]. Symptoms include dysphonia (hoarse voice), tongue deviation towards the affected side, lingual motility disturbance, ipsilateral vocal cord paralysis and swallowing difficulty [41, 42]. Although rare, the most frequent reported cause of Tapia's syndrome is orotracheal intubation during general anaesthesia or prolonged intubation and assisted ventilation in the intensive care setting where the nerves can be compressed and/or stretched [1, 43]. Neck trauma and manipulations, including rapid neck rotations, improper neck positioning during general anaesthesia, cervical spine surgery, an enlarged hyoid bone and vertebral or carotid artery dissection, have also been proposed as other causes of combined HN and RLBVN injury and the subsequent syndrome [13, 41, 43]. Although both the intracranial and extracranial portions of the HN can be impacted, the extracranial HN, where it and the RLBVN lie in close proximity, is the most common reported site affected [43].

Radiation induced HN fibrosis and subsequent palsy can result from radiation therapy for treatment of head and neck cancers, particularly nasopharyngeal carcinoma [6]. Acute radiation nerve injury, occurring during months after treatment, is usually reversible and presents as nerve thickening on imaging [37]. Radiation neuropathy usually peaks 1–2 years after treatment [6]. HN palsy, however, is more commonly reported in a more delayed (2–10 years) post radiation treatment, which can result in highly debilitating permanent late complications, including profound impacts on speech and swallowing, late radiation associated dysphagia, leading to a lifetime dependence on non‐oral feeding [13, 37, 44, 45]. Delayed radiation HN neuropathy is most commonly due to nerve entrapment/compression by fibrosis [37].

The HN can be focally compressed and entrapped by surrounding neck pathology or injury. Cervico‐cerebral arterial dissections (CAD), reported to contribute to 20% of strokes in people under 45 years of age, can result in HN compression intracranially [46]. The close proximity of the HN to the internal carotid artery (ICA) can make the HN vulnerable to compression due to ICA dissections and subsequent dilatation [47]. Trauma‐induced CAD can be caused by motor vehicle accidents, neck manipulations and prolonged general anaesthesia during surgical procedures and strangulations, both fatal and non‐fatal [47, 48, 49, 50].

The extracranial HN can be compressed due to IJV or EJV thrombus and aneurysmal carotid arteries [13]. Fractures of the occipital condyle can result in haematoma or bone fragments or bony callous that can compress or irritate the HN [1]. This can result in symptoms including pain in the unilateral upper neck and/or posterior head and pain in one side of the tongue termed neck‐tongue syndrome [5].

HN extrinsic compression can also be due to tumours around its path including squamous cell carcinoma, lymphoma, salivary gland malignancies, sarcomas, enlarged lymph nodes or lipomas, neck abscesses or infections [2]. The HN may also be directly impacted by tumour infiltrating the nerve [51]. HN tumours can be located intracranially and/or extracranially and symptoms can vary depending on the location of the tumour; tongue disturbances are identified in most patients; however, occipital headache exacerbated by neck movement is also a symptom [51].

## 9 Planning Ultrasound Guidance for Interventions Around the HN

Ultrasound imaging can be used to guide extracranial HN blocks or radiofrequency (RF) ablations. Perineural HN nerve block injections can allow for hydrodissection and relief of areas of HN entrapment and associated symptoms. Ultrasound guided HN blocks in the sublingual space can be used to facilitate incisional tongue biopsies and the excision of benign tongue lesions [52]. For ultrasound guided HN nerve blocks, the needle can be guided from a posterior to anterior approach to enable access to the horizontal HN. Ultrasound imaging can also allow monitoring of nerve recovery or disease progression [19]. Ultrasound guided stimulation of the HN and ansa cervicalis can be used in patients with obstructive sleep apnoea (OSA) [5, 28]. Stimulation of the HN can result in increasing tone in the genioglossus muscle and assist in maintaining pharyngeal patency during sleep [5, 28].

## 10 Conclusion

Injury, compression, entrapment, or lesions of the HN can occur at any point along its path and cause HN palsy. MRI or CT is used when investigating the intracranial and skull base segments of the HN and its surrounding soft tissues and bony structures. The extracranial HN can be imaged sonographically, and relevant anatomical landmarks are required to be used to allow correct identification of the HN and demonstration of any associated injury, entrapment, or pathology, or associated muscle denervation. Studies investigating the sonographic size of the HN are required.

## Ethics Statement

The authors have nothing to report.

## Conflicts of Interest

The authors declare no conflicts of interest.

## Supporting information


Appendix S1.

## Data Availability

Data sharing not applicable to this article as no datasets were generated or analysed during the current study.
