# Supplementary material for: Sonographic Anatomy and Imaging of the Extracranial Component of the Hypoglossal Nerve (CNXII)
Source: J Med Radiat Sci. 2025 Jul 18;72(4):417–29. doi: 10.1002/jmrs.70010 (PMC12661075; doi:10.1002/jmrs.70010)
Supplement: Supplementary file 1 — Appendix S1. [file JMRS-72-417-s001.zip › Place holder image.docx]

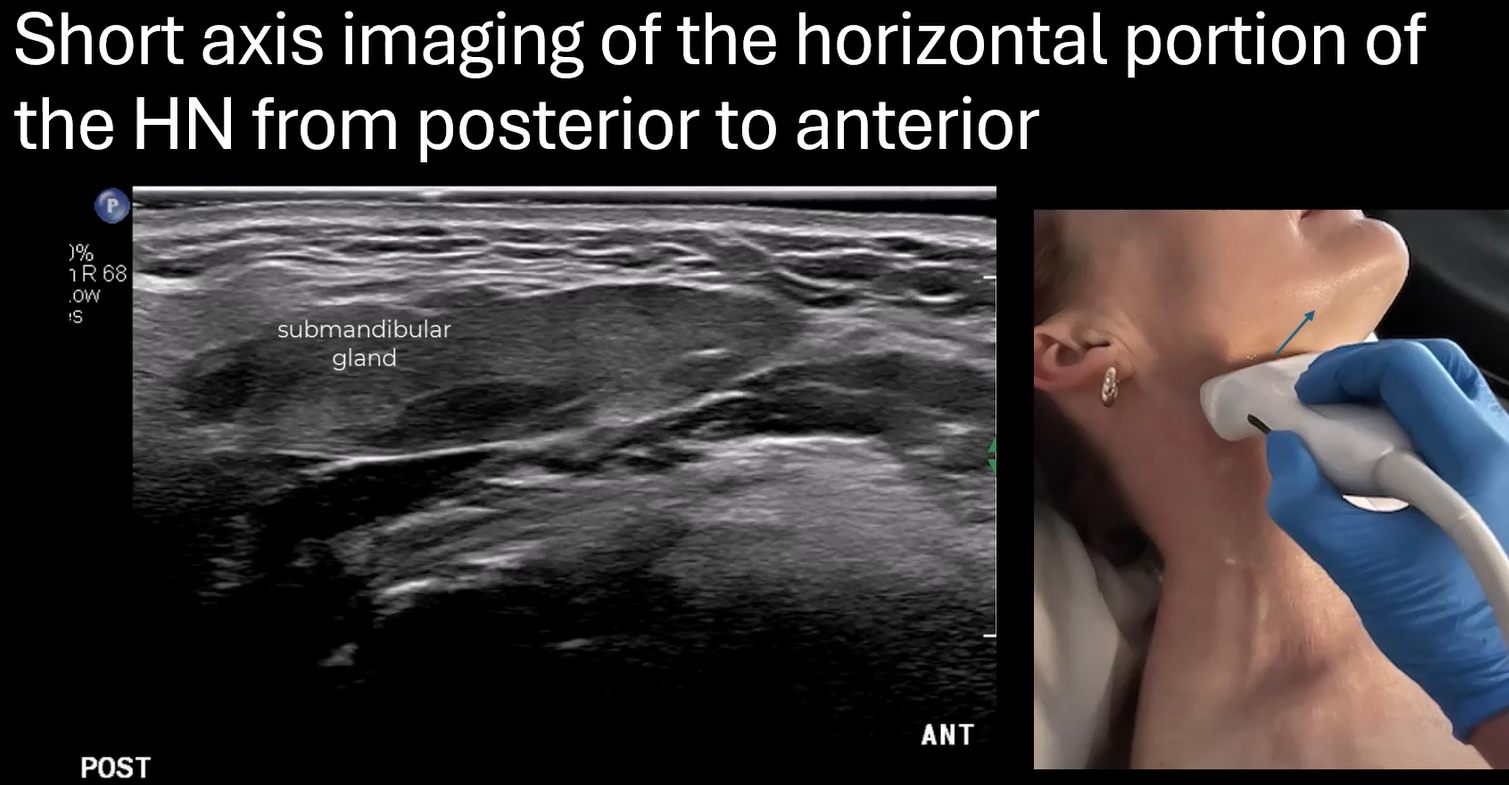


*Video 1. Short axis sonographic imaging of the hypoglossal nerve.*


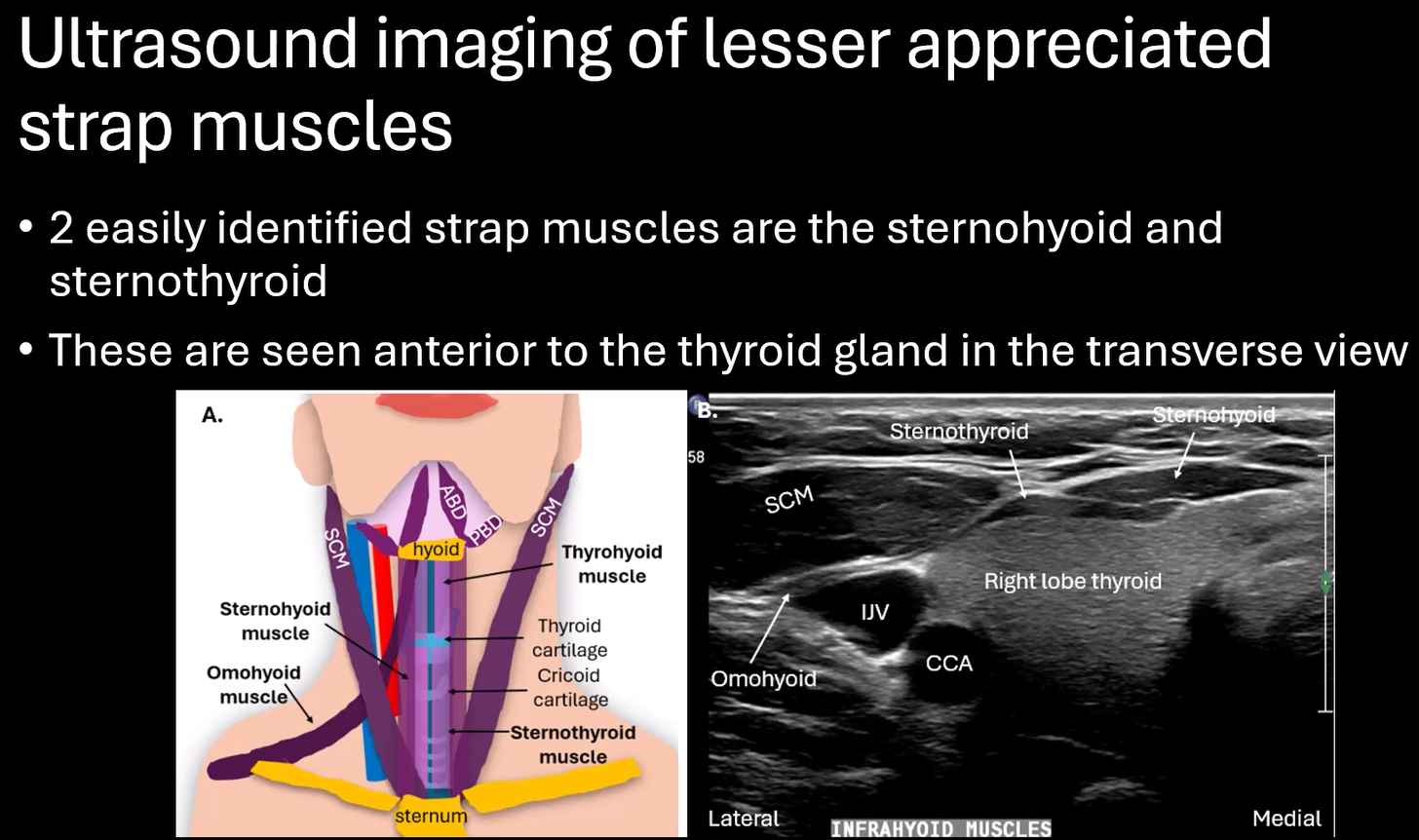


*Video 2. Ultrasound imaging of the omohyoid and thyrohyoid muscles.*
